# IVIG regulates the survival of human but not mouse neutrophils

**DOI:** 10.1038/s41598-017-01404-0

**Published:** 2017-05-02

**Authors:** Christoph Schneider, Simone Wicki, Stefanie Graeter, Tankica M. Timcheva, Christian W. Keller, Isaak Quast, Danila Leontyev, Iglika K. Djoumerska-Alexieva, Fabian Käsermann, Stephan M. Jakob, Petya A. Dimitrova, Donald R. Branch, Richard D. Cummings, Jan D. Lünemann, Thomas Kaufmann, Hans-Uwe Simon, Stephan von Gunten

**Affiliations:** 1 Institute of Pharmacology, University of Bern, Bern, Switzerland; 2 Institute of Experimental Immunology, Laboratory of Neuroinflammation, University of Zurich, Zurich, Switzerland; 3 Department of Immunology and Pathology, Central Clinical School, Monash University, Melbourne, Australia; 4 Department of Medicine, University of Toronto and Centre for Innovation, Canadian Blood Services, Toronto, Ontario Canada; 5 Department of Immunology, Stefan Angelov Institute of Microbiology, Bulgarian Academy of Sciences, Sofia, Bulgaria; 6 CSL Behring, Research, Bern, Switzerland; 7 Department of Intensive Care Medicine, University Hospital Bern (Inselspital), University of Bern, Bern, Switzerland; 8 Department of Surgery, Beth Israel Deaconess Medical Center, Harvard Medical School, Boston, MA USA

## Abstract

Intravenous immunoglobulin (IVIG) are purified IgG preparations made from the pooled plasma from thousands of healthy donors and are being tested in preclinical mouse models. Inherent challenges, however, are the pluripotency of IVIG and its xenogeneicity in animals. IVIG can alter the viability of human neutrophils via agonistic antibodies to Fas and Siglec-9. In this study, we compared the effects of IVIG on human and mouse neutrophils using different death assays. Different commercial IVIG preparations similarly induced cytokine-dependent death in human neutrophils, whereas they had no effects on the survival of either peripheral blood or bone marrow neutrophils from C57BL/6 or BALB/c mice. F(ab’)_2_ but not Fc fragments of IVIG induced death of human neutrophils, whereas neither of these IVIG fragments, nor agonistic monoclonal antibodies to human Fas or Siglec-9 affected the viability of mouse neutrophils. Pooled mouse IgG, which exhibited a different immunoprofile compared to IVIG, also had no effect on mouse cells. Together, these observations demonstrate that effects of IVIG on neutrophil survival are not adequately reflected in current mouse models, despite the key role of these cells in human inflammatory and autoimmune diseases.

## Introduction

Intravenous immunoglobulin (IVIG) preparations consist primarily of IgG antibodies produced from pooled plasma from thousands of healthy donors. Initially used for the antibody replacement therapy of humoral immunodeficiencies, to date their value as anti-inflammatory drugs is appreciated for the treatment of a steadily increasing number of disorders across medical disciplines, including rheumatology, neurology, dermatology, gynecology, and transplantation medicine^1–4^. Given their polyclonal nature, these immunoglobulin preparations contain a wide range of specificities for antigens^5^, reflecting the combined antibody repertoire of the donor population^6^. Within this repertoire functional antibodies with immunomodulatory capacity have been identified that target virtually all arms of the humoral and cellular immune systems, and include immunoregulatory, neutralizing, or anti-idiotypic antibodies^1, 6–9^.

Animal models revealed immunomodulatory antibodies in IVIG that ameliorate the course of autoimmune disorders, such as lupus, myasthenia gravis, pemphigus vulgaris and antiphospholipid syndrome (APS) by targeting specific pathogenetic mechanisms^10^. Based on these *in vivo* experiments, it was proposed that specific IVIG (sIVIG) enriched for the active compounds may have an advantage over regular IVIG^10^. Other studies suggest that sialylation of both the Fc and Fab regions of IgG may contribute to the anti-inflammatory effects of IVIG^11–13^; yet, conflicting evidence in models of immune thrombocytopenia (ITP) and rheumatoid arthritis (RA) indicates the need for further investigations^3, 14–16^, in which potential experimental limitations related to the disease model or to intrinsic characteristics of IVIG are given special attention in terms of study design and data interpretation^17, 18^. The pharmacological complexity of IVIG is determined by its pluripotency^6, 17, 19^, polyclonality, and origin from different individuals. Notably, these complex human preparations might have xenogeneic effects at least on certain immunological players in animals, eventually leading to loss-of-function or gain-of-function effects^7^. Species-related differences in IVIG functions might be common^20^. It is, therefore, imperative for the design and interpretation of future *in vivo* studies on IVIG to dissect species-related similarities and differences of potential IVIG targets.

Neutrophils are key players of innate immunity and often the most predominant leukocyte at the site of inflammation, in particular at acute stages of autoimmune or other inflammatory disorders^21–23^. Upon activation, these cells cannot only cause substantial tissue damage, but recent evidence suggests that neutrophils play an active role in the coordination of innate and adaptive immunity^24, 25^. In humans, but not in mice, neutrophils represent the most frequent leukocytes in the circulation. Neutrophils are short-lived cells and the regulation of neutrophil survival is considered as a mechanism to control this innate effector cell^26–28^. Clinically relevant concentrations of IVIG can regulate the survival of neutrophils in a cytokine-dependent manner^29, 30^. Functional antibodies to the death receptor Fas (also called CD95) and to Siglecs have been implicated in the regulation of granulocytes by IVIG^9, 29–32^. In mouse neutrophils, IVIG was reported to limit inhibition of neutrophil apoptosis induced by lipopolysaccharide (LPS) stimulation, potentially by blocking LPS-mediated effects, although no direct pro-apoptotic activity of IVIG could be demonstrated^33^.

Here we report our discovery that different commercial IVIG preparations equally induce cytokine-dependent death of human neutrophils, whereas mouse neutrophils, regardless of cytokine priming or origin from bone marrow or circulation, were resistant to IVIG-induced death. Unexpectedly, we observed no signs of cell death in mouse neutrophils following IVIG treatment, neither in terms of genomic DNA fragmentation, phosphatidylserine (PS) redistribution, cell permeability, loss of mitochondrial membrane potential, nor morphology. Human neutrophil death was triggered in a F(ab’)_2_ but not Fc-dependent manner, supporting the notion that specific antibodies in IVIG are responsible for granulocyte death in humans. Mouse neutrophils were also resistant to homologous pooled IgG from mice, indicating that the use of species-matched pooled IgG may not provide an adequate alternative to mimic IVIG effects in animal models. These findings suggest that despite the key role of neutrophils in autoimmune disease and other inflammatory disorders, currently used mouse models may not reflect important effects of IVIG on human neutrophils.

## Results

### Different effects of IVIG on human versus mouse neutrophil survival

**Figure 1:**
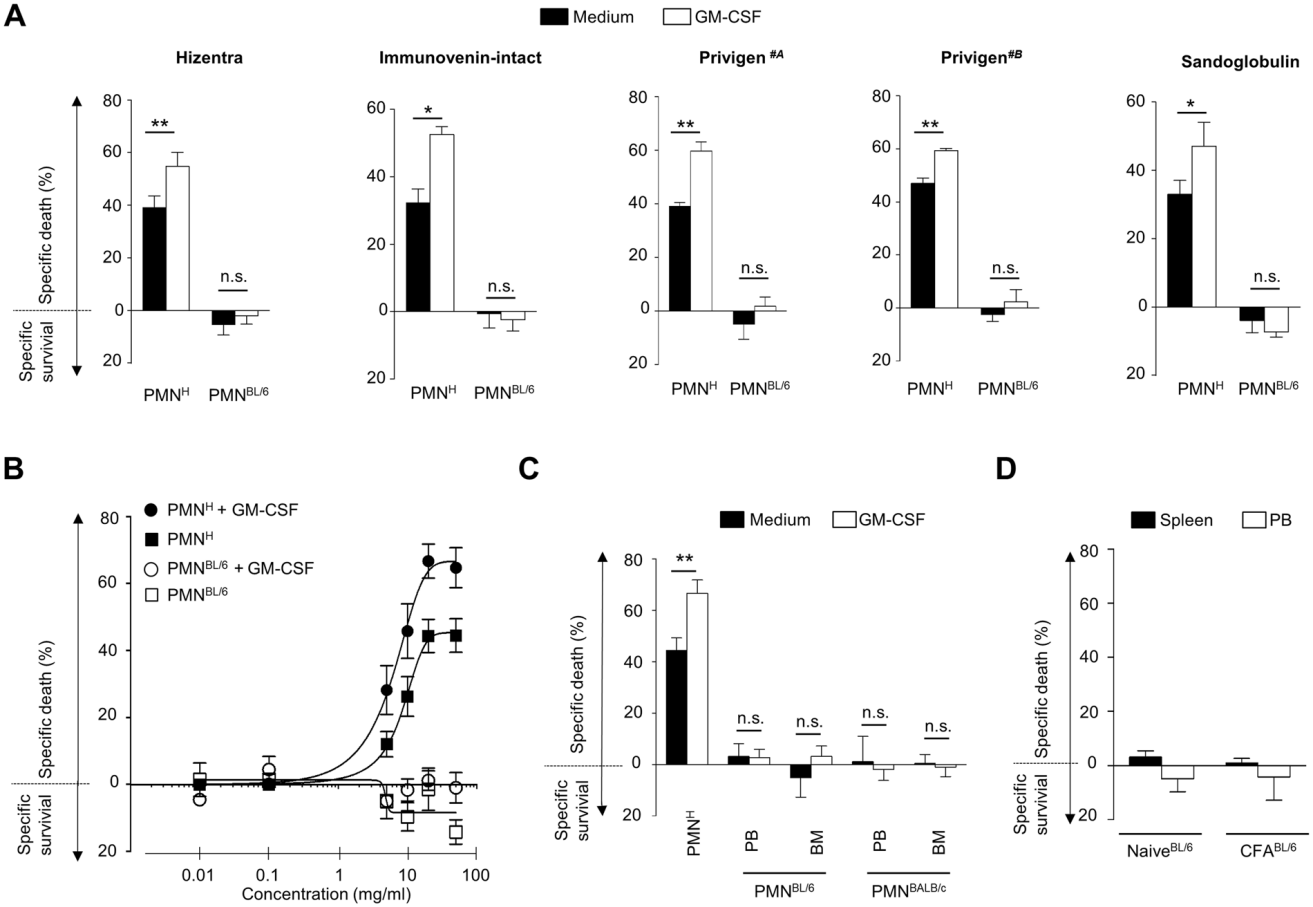
IVIG induces cell death in human but not in mouse polymorphonuclear neutrophils (PMNs) *ex vivo*. Death of human (PMN^H^), C57BL/6 (PMN^BL/6^) or BALB/c (PMN^BALB/c^) mouse neutrophils in the presence or absence of priming with species matched GM-CSF, as assessed by flow cytometric ethidium bromide exclusion assay. (**A**) Different commercial IVIG/SCIG preparations similarly induce death of human peripheral blood neutrophils, an effect that is enhanced in GM-CSF primed cells. No death is induced in mouse PB neutrophils. (**B**) Comparison of the concentration-effect curves of IVIG in PB PMN^H^ or PMN^BL/6^ with or without GM-CSF priming. (**C**) In mouse neutrophils from both strains, no IVIG-mediated death is observed, neither in cells derived from peripheral blood (PB) nor bone marrow (BM). (**D**) In *in vivo* primed neutrophils isolated from spleen or peripheral blood (PB) no IVIG-induced cell death was observed. Results of 24-h cultures are shown. Bars show mean ± SEM. Data are representative of at least 3 (**D**), 4 (**C**), 5 (**B**), or 6 (**A**) independent experiments. *p < 0.05, **p < 0.01, Student *t* test. Specific death was calculated in comparison to untreated controls as outlined in the Materials and Methods section.

IVIG can regulate the survival of human neutrophils, an effect that is enhanced in cells under inflammatory conditions^29–31^. We compared the efficacy of different lots or batches of commercial IVIG preparations to modulate the survival of human and mouse neutrophils. Hizentra, Immunovenin-intact, Sandoglobulin, and two lots of Privigen, all at 20 mg/ml, a concentration that following high-dose IVIG treatment is typically reached in the circulation^34^, induced cell death in human peripheral blood neutrophils to a similar extent, and this effect was further enhanced following priming of cells with human GM-CSF (Fig. 1A). In contrast, none of the preparations had an effect on the survival of peripheral blood neutrophils of mice, either in the presence or absence of mouse GM-CSF. In line with previously published results^29, 31^, the effects of IVIG on human neutrophil survival were concentration-dependent, whereas we observed no death-induction on mouse neutrophils even at high concentrations (Fig. 1B). We also investigated the responses of mouse cells from different compartments, but found no death responses to IVIG in either peripheral circulatory or in the less mature bone marrow-derived mouse neutrophils from both C57/BL6 and BALB/c mouse strains, respectively (Fig. 1C, Supplementary Fig. 1). Further, when C57/BL6 mice were immunized with complete Freund’s adjuvant (CFA), which is known to induce strong neutrophil responses^35, 36^. The activation marker CD11b was upregulated on neutrophils isolated from spleen (Supplementary Fig. 2), but no IVIG-induced neutrophil cell death was observed in neutrophils isolated from spleen or peripheral blood (Fig. 1D). Peritoneal injection of IVIG up to 4 g/kg in healthy BALB/c mice did not affect peripheral blood neutrophil counts *in vivo* nor flow cytometric Annexin V staining (Supplementary Fig. 3), a marker for apoptosis. Furthermore, intravenous IVIG administration did not alter the frequency of Annexin V^+^ blood neutrophils in BALB/c mice with LPS-induced sepsis (Supplementary Fig. 3). Interestingly, non-rodent peripheral blood neutrophils *ex vivo* from a porcine sepsis model of fecal peritonitis also showed no death response in 24-h cultures in the presence of IVIG (Supplementary Fig. 4).

### Occurrence of IVIG-induced features of cell death in human but not mouse neutrophils

**Figure 2:**
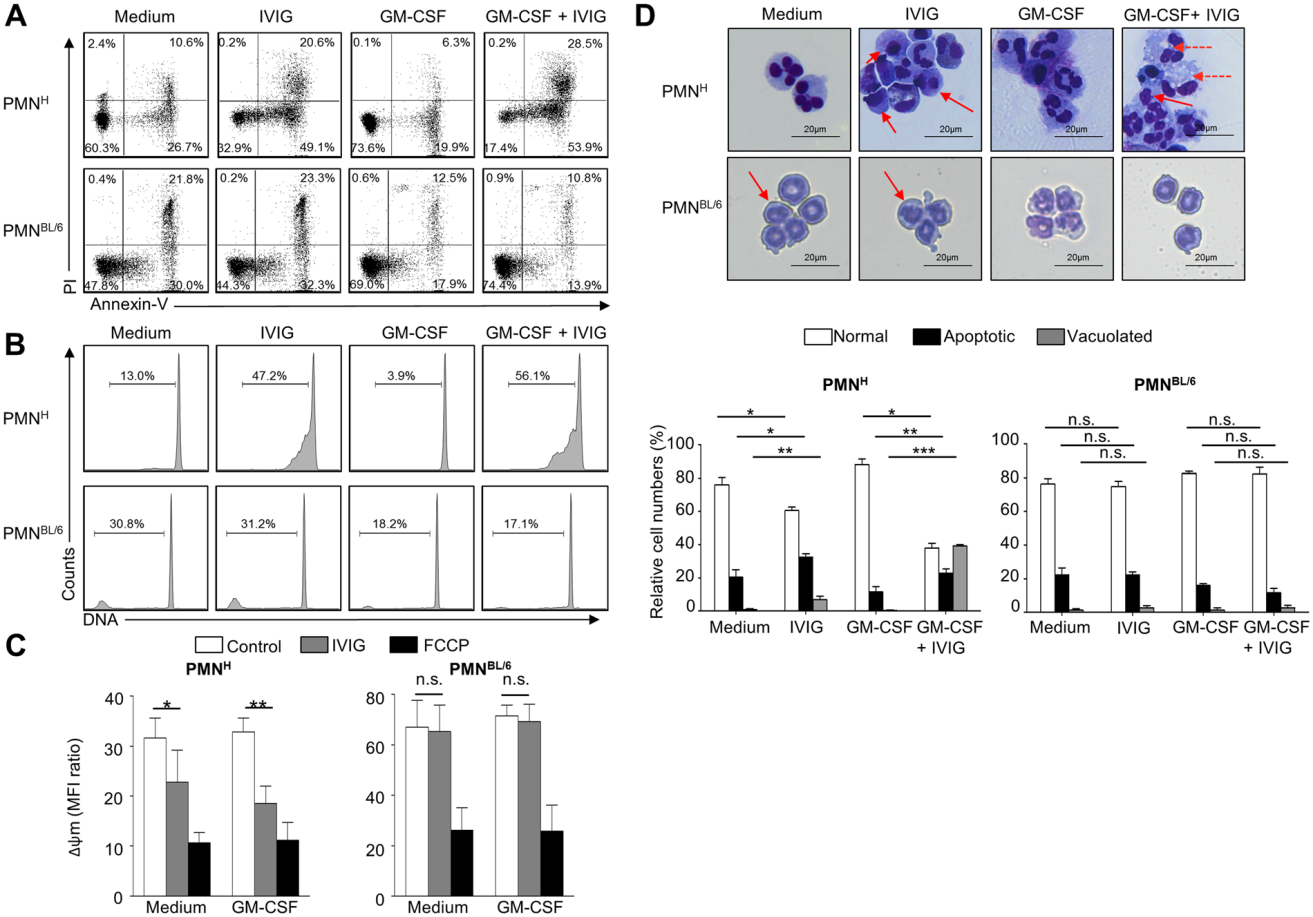
Confirmation of selective survival regulatory capacity of IVIG in human but not mouse neutrophils by different death assays. Features of cell death assessed in GM-CSF primed or unprimed human (PMN^H^) or C57BL/6 mouse (PMN^BL/6^) neutrophils following treatment with IVIG by flow cytometry (**A**–**C**) or light microscopy (**D**). (**A**) Annexin V-FITC/PI staining assay. Quantitative analysis is indicated as the percentage representative of each quadrant. (**B**) DNA-fragmentation assay. Quantitative analysis of gated subdiploid DNA is indicated. (**C**) Assessment of mitochondrial potential (Δψm). The mitochondrial uncoupler FCCP was used as a positive control. Results of 5- (**C**) or 15-hour cultures (**A**,**B**,**D**) are shown. (**D**) Morphological characterization. Cells were stained with Giemsa–May–Grünwald (Diff-Quik) (original magnification X1000). Continuous arrows indicate condensed nuclei, dashed arrows vacuoles. The lower panel represents the statistical analysis of the normal, apoptotic or vacuolated phenotypes. Data are representative for at least 3 independent experiments. Bars show mean ± SEM. *p < 0.05, **p < 0.01, one way-ANOVA followed by Dunnet’s post hoc test. Δψm = mitochondrial potential.

To further investigate the susceptibility of mouse neutrophils toward IVIG-induced death effects, we analyzed various markers of cell death. As expected for a sign of spontaneous apoptosis, phosphatidylserine (PS) redistribution to the surface was evident in aged human and mouse neutrophil cultures (15 hours) by flow cytometric Annexin V staining, which was delayed in the presence of species-matched GM-CSF (Fig. 2A, Supplementary Figs 5 and 6). IVIG treatment increased the frequency of Annexin V+ human neutrophils, and this was further enhanced by GM-CSF priming. In contrast, IVIG had no effects on either PS redistribution or on propidium iodide uptake of mouse neutrophils compared to cytokine-primed or unprimed controls. Similarly, IVIG treatment led to increased DNA fragmentation exclusively in human cells (Fig. 2B, Supplementary Fig. 6). We also could detect dissipation of the mitochondrial membrane potential (Δψm) in human neutrophils following IVIG treatment for 5 hours as a sign of early mitochondrial apoptosis pathway activation (Fig. 2C), whereas there was no effect of IVIG on Δψm in mouse neutrophils upon treatment with IVIG.

Morphological characterization by light microscopy revealed signs of spontaneous apoptosis, as expected, in 15-hour cultures of both mouse and human neutrophils. The frequency of cells with an apoptotic phenotype (cellular shrinkage, nuclear condensation) was enhanced in IVIG-treated human neutrophils, and extensive cytoplasmic vacuolization appeared in treated cells in the presence of GM-CSF (Fig. 2D), an observation that has been associated with neutrophil death by IVIG^29^, Siglec-9^37^, or CD44^38^, under inflammatory conditions. By contrast, we observed no signs of increased cell death or cytoplasmic vacuole formation in mouse neutrophils treated with IVIG. Together, these findings indicate that IVIG triggers apoptotic pathways in human but not in mouse neutrophils.

### IVIG-mediated death effects on human neutrophils are F(ab’)_2_- but not Fc-mediated

**Figure 3:**
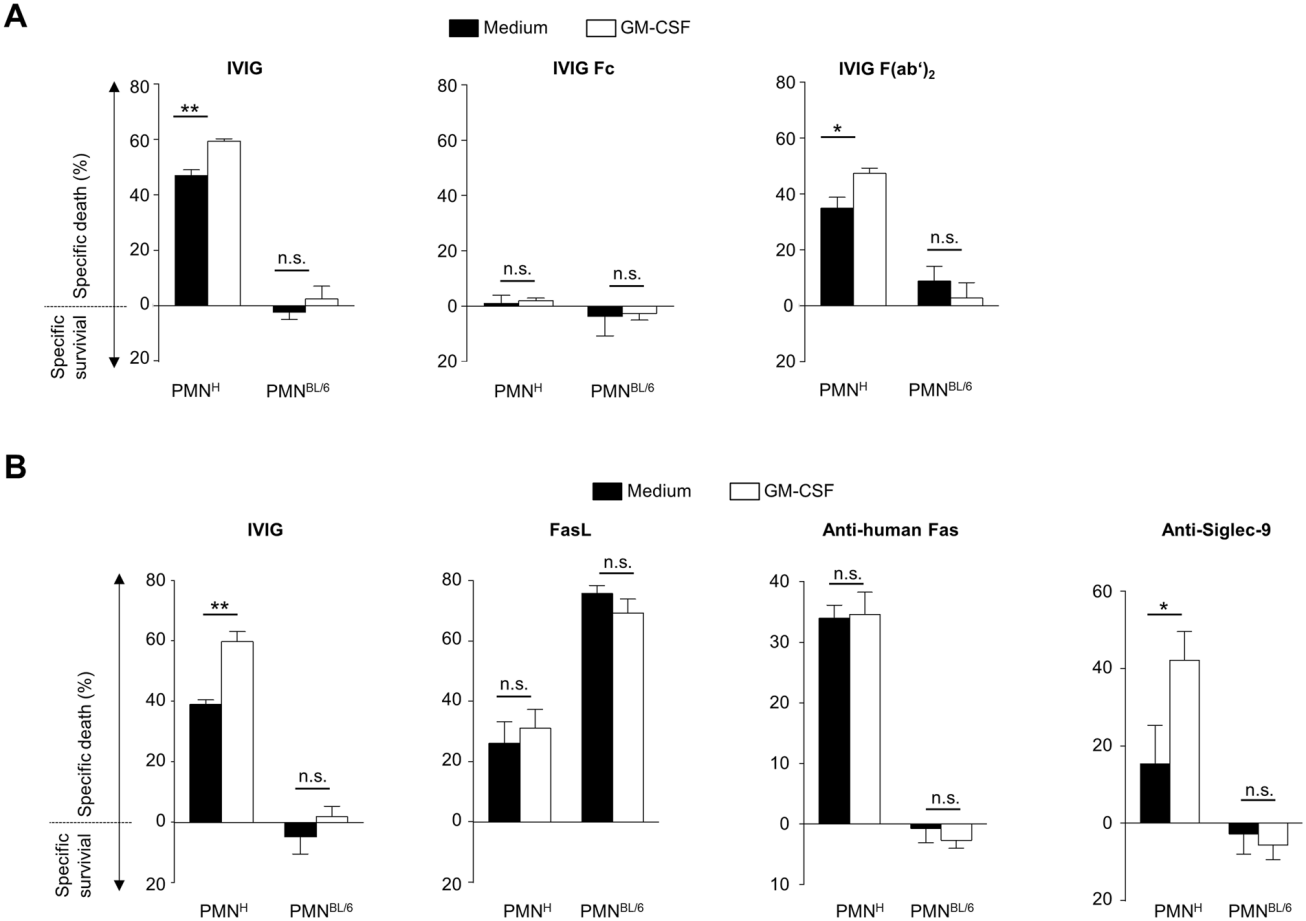
Human neutrophil susceptibility to death stimuli is specific, Fc-independent, and differs from responses of mouse neutrophils. Death of human (PMN^H^) or mouse C57BL/6 (PMN^BL/6^) polymorphonuclear neutrophils as assessed by flow cytometric ethidium bromide exclusion assay. Results of 24-h cultures of quiescent or GM-CSF primed cells are shown following treatment with (**A**) 20 mg/ml of whole IgG (Privigen^#B^) or equimolar concentrations of Fc-, or F(ab’)_2_ (Gammavenin)-fragments of pooled IgG, or with (**B**) stimuli known to trigger human neutrophil death including IVIG (Prigiven^#A^), Fas-ligand, anti-human Fas (clone CH11) mAb or anti-Siglec-9 mAb. Bars show mean ± SEM. Data are representative of at least 3 (**B**), or 4 (**A**) independent experiments. *p < 0.05, **p < 0.01, Student t test. Specific death was calculated in comparison to untreated controls as outlined in the Materials and Methods section.

The implication of Fc- versus specific Fab-mediated effects of IVIG on the regulation of different leukocytes remains to be explored^1, 17, 29^. Therefore, we investigated the effects of whole IVIG with Fc-, or F(ab’)_2_ (Gammavenin)-fragments of pooled IgG on human and mouse neutrophils. The streptococcal cysteine proteinase Ide-S was used to generate Fc and F(ab’)_2_ fragments from IVIG^39^. In line with previous findings^29, 30^, IVIG and the preparation of F(ab’)_2_-fragments both induced death of human neutrophils that was enhanced in GM-CSF primed cells (Fig. 3A). In contrast, we did not observe any effects of Fc fragments of IVIG on the survival of neutrophils. In addition, neither Fc-, nor F(ab’)_2_ preparations affected mouse neutrophils.

The observed findings contribute to accumulating evidence that IVIG mediates the regulation of neutrophil survival by antibodies with specificity to human-specific antigenic determinants. Functional antibodies to Fas and Siglec-9 are thought to be responsible for the regulatory effects of IVIG on the survival of human neutrophils^29, 30^. Commercial agonistic antibodies to human Fas or Siglec-9 receptors, like IVIG, induced death in human but not mouse neutrophils, whereas mouse neutrophils were highly responsive to crosslinked Fas ligand (FasL), in accordance with published reports^40^ (Fig. 3B). Induction of death in human neutrophils by anti-Siglec-9 monoclonal antibody (mAb) was GM-CSF-dependent, which supports the possibility that the cytokine-dependent effects of IVIG might be linked to targeting of Siglec-9^29^.

### Pooled IgG from mouse sera does not induce death in autologous neutrophils

**Figure 4:**
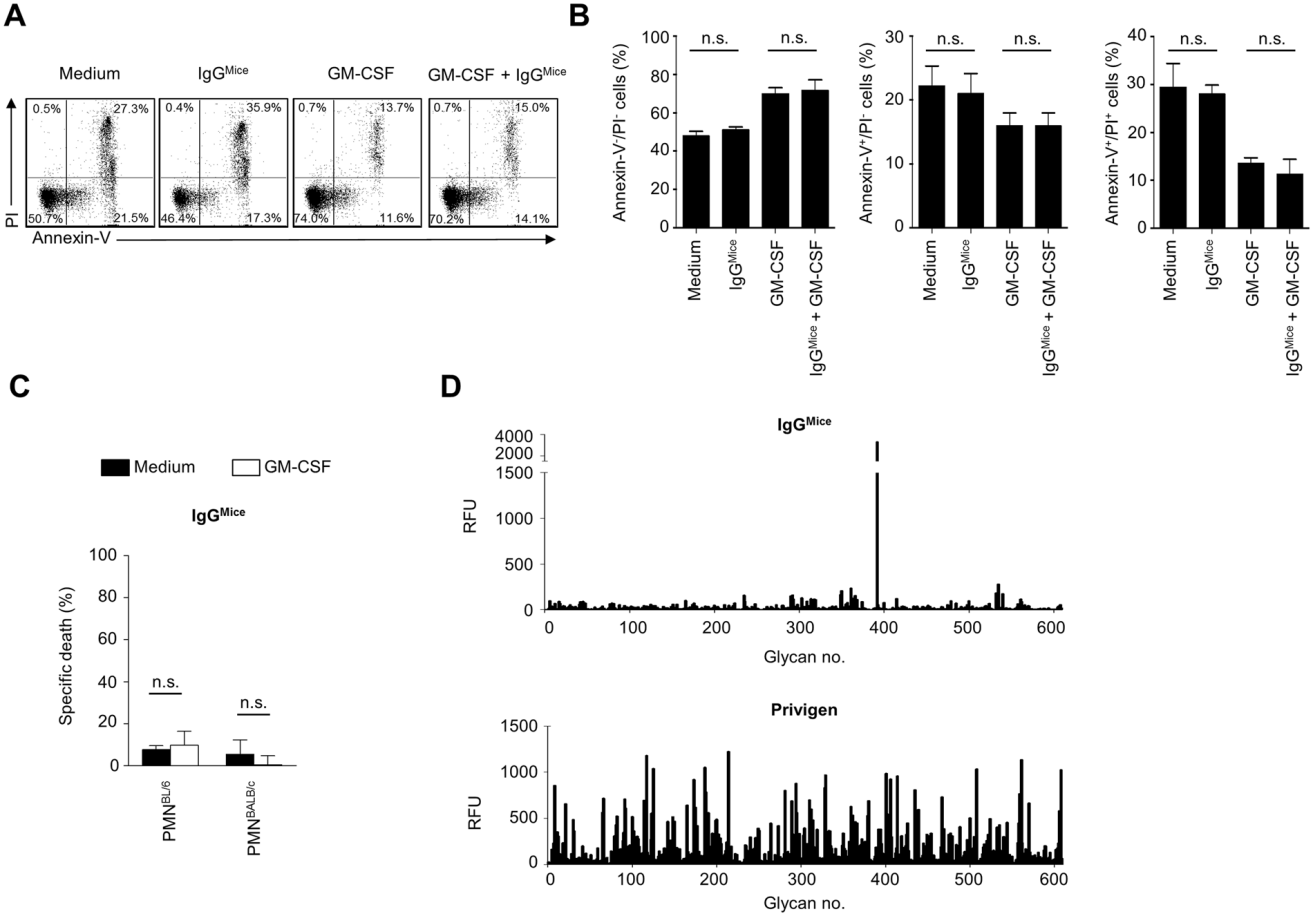
Mouse polyclonal IgG does not induce cell death in mouse neutrophils. Flow cytometric characterization of C57BL/6 mouse neutrophil cell death of unprimed or primed cells by mouse polyclonal IgG (IgG^Mice^). Representative example of Annexin V-FITC/PI staining assay (**A**) and statistical summaries (**B**) of Annexin^−^/PI^−^-, Annexin^+^/PI^−^- and Annexin^+^/PI^+^-gated cell populations are indicated for analysis after 15 h incubation with polyclonal mouse IgG. (**C**) Death of mouse C57BL/6 mouse (PMN^BL/6^) or BALB/c mouse (PMN^BALB/c^) neutrophils after IgG stimulation as assessed by ethidium bromide exclusion assay. (**D**) Binding profiles of IgG^Mice^ or IVIG to 609 glycan antigens, as assessed by glycan array technology. RFU = relative fluorescence units. Bars show mean ± SEM (n = 3). *p < 0.05. One way-ANOVA followed by Dunnets posttest. Specific death was calculated in comparison to untreated controls as outlined in the Materials and Methods section.

IVIG preparations consist primarily of IgG derived from the sera of thousands of donors and hence reflect the IgG antibody repertoire of the donor population. Recently, it was reported that the specific recognition of antigens by IgG antibodies varies substantially between sera from different species^41^. Given that IVIG-induced death in human neutrophils depends on F(ab’)_2_-fragments that are responsible for antigen recognition, we wondered if pooled IgG from mouse sera would also have the capacity to trigger death in neutrophils of the same species (mice). Mouse neutrophils were treated with commercial IgG derived from pooled mouse sera at 20 mg/ml and cell death of 15-hour cultures was assessed by annexin V-FITC/PI staining and subsequent flow cytometric analysis. Pooled mouse IgG had no effect, neither on PS redistribution nor PI incorporation of unprimed or GM-CSF primed cells (Fig. 4A and B). Glycan array technology, a powerful tool to assess immunoprofiles^5, 41^ was used to screen the reactivity of pooled mouse IgG with IVIG to 609 glycan antigens. While human IVIG bound a broad range of glycans on the array, recognition by pooled mouse IgG was restricted to only few blood group antigens that are also found in bacteria, including GalNAcα1-3(Fucα1-2)Galβ1-3GalNAcα1-3(Fucα1-2)Galβ1-4GlcNAc (Supplementary Fig. 7). These findings indicate that pooled mouse sera exhibit a significantly different immunoprofile compared to IVIG, and do not contain functional antibodies with the capacity to regulate the survival of autologous neutrophils.

## Discussion

In humans, neutrophils are the most frequent nucleated cells in the circulation (50–70% neutrophils) and are involved in the pathogenesis of a wide range of inflammatory and autoimmune disorders^23–25^. Compared to their human counterparts, mouse neutrophils are significantly less frequent in the blood (75–90% lymphocytes, 10–25% neutrophils)^42^, and also exhibit functional differences, e.g. in terms of the expression of immunological effector molecules^43^. In both species, neutrophil clearance by death is considered an efficient means to control this cell type^26, 28, 44^, which also represents one pharmacodynamic effect of IVIG on human neutrophils^9, 29–32^. We^11, 39, 45^ and others^1, 4^ have tested human IVIG in xenogeneic animal models and gained significant mechanistic insights. However, in the present work we show that mouse neutrophils are resistant to IVIG-mediated cell death, indicating that animal models do not show, or, potentially, even provide misleading information on the important effects of IVIG on neutrophils and neutrophil-modulated processes^24, 25^ that are relevant for the pathogenesis of human disease.

We observed that IVIG induced death of human neutrophils in a cytokine-dependent manner, a finding that is in line with previous publications^29, 30^. Similarly, clinical studies reported decreased peripheral neutrophil counts after IVIG-treatment in patients suffering from ITP^46^ or neuroimmunologic disorders^47^. The augmented sensitivity of human neutrophils under inflammatory conditions, and thus increased IVIG potency, might be beneficial in terms of primarily targeting neutrophils at the site of inflammation. This phenomenon might also explain the relative sparing effect on resting neutrophils (yet, transient neutropenia is sometimes observed upon IVIG treatment^7, 48^), dependence of IVIG efficacy on disease stage, and the pharmacodynamics efficacy of specific antibodies at low frequency in IVIG^5^. Previous reports showed that death-promoting effects of IVIG on human neutrophils are F(ab’)_2_-mediated^29, 30^, and involve the action of agonistic antibodies to Fas and Siglec-9 receptors^29–31^, whereby the cytokine-dependent effects seem to be mediated by functional anti-Siglec-9 antibodies in IVIG^29^. Further, several studies associated the effects of IVIG on human neutrophils with an enhancement of autophagic activities^49–51^.

Both, quiescent and primed neutrophils are known to express the Fc receptors CD16, CD32 and CD64^52^. Here, we provide a novel insight that Fc-fragments of IVIG have no effect on neutrophil viability, supporting the notion that IVIG-mediated neutrophil death relies entirely on specific functional antibodies rather than on unspecific or Fc-γ receptor dependent mechanisms. In contrast to the effects on human neutrophils, neither total IVIG nor its F(ab’)_2_- or Fc-fragments affected survival of peripheral blood or bone marrow neutrophils derived from C57BL/6 or BALB/c mice. Despite evidence of IVIG-binding to self-antigens^53^, to our knowledge no reports show clinically relevant tissue-destruction related to autoreactivity by antibody-dependent cellular cytotoxicity (ADCC) following IVIG administration.

Commercial IVIG preparations, including Immunovenin-intact, Hizentra, Sandoglobulin and two different lots of Privigen, all similarly induced cytokine-dependent death of human, but not mouse, neutrophils. Absence of IVIG-mediated death was confirmed in different death assays, including flow cytometric assessment of Annexin-V/PI staining or ethidium bromide exclusion, DNA fragmentation, mitochondrial potential measurement, and morphological analysis. These data demonstrate that IVIG mediates death of human neutrophils by the action of specific antibodies directed to targets that are recognized and functional in human, but not in mouse neutrophils.

Treatment with Siglec-9-specific mAb resulted in cytokine-dependent death of human neutrophils, but expectedly had no effect on mouse neutrophils on which this receptor is absent. The functional orthologue of human Siglec-9 in mice is Siglec-E^54^, but it remains to be shown if this receptor is capable to activate death pathways in mouse neutrophils. Fas is expressed in both species, and mouse neutrophils were highly susceptible to Fas ligand-mediated death. In contrast, a human-specific anti-Fas mAb induced human neutrophil death, but failed to induce apoptosis in mouse cells. It is likely that the clone differentially recognized antigenic structural determinants on human and mouse Fas, but might also be related to subtle differences in proximal signaling events. These data illustrate how specific antibodies to known targets of IVIG, i.e. Fas and Siglec-9^29–31^, are functional on human neutrophils, but dysfunctional on mouse neutrophils, due to species-related differences in receptor expression, signaling or cellular biology.

To avoid species-related bias of xenogeneic IVIG we additionally tested the susceptibility to polyclonal IgG-induced neutrophil cell death in a mouse analogue, i.e. a mouse polyclonal preparation of pooled IgG antibodies (IgG^Mice^), as a substitute for studies in mice. However, IgG^Mice^ did not promote death in neutrophils of C57BL/6 nor BALB/c mice. Glycan array technology has previously been used to assess immunoprofiles of IVIG preparations^5, 55–58^, and has recently revealed species-related differences in antigen recognition patterns of sera by Stowell *et al*.^41^. Using this technology we observed that the immunoprofiles of IgG^Mice^ and (human) IVIG differed dramatically. Given these species-related differences in specificities, as well as the known differences in antibody isotypes and Fc-receptors^59^, the use of IgG^Mice^ in animal models appears inadequate as a substitute for xenogeneic IVIG for the study of neutrophil-dependent or –independent immune responses.

In 2004 Mestas and Hughes described differences between mouse and human immunology, and highlighted a tendency to ignore differences in experimental models with the associated risk of overlooking aspects of human immunology that do not occur, or cannot be modeled, in mice^59^. Indeed, such differences have led to many controversies in different areas of experimental immunology, including B cell biology^60, 61^, models of inflammatory diseases^62, 63^, immune thrombocytopenia^18^, or multiple sclerosis (MS)^59, 64^. In many cases, protective effects of agents observed in animal models had no effects in the clinics, as for IVIG treatment in MS^64^, or even led to disease exacerbation, as observed for IFN-γ in MS^65^, or to unexpected, even life-threatening, immune reactions in humans, as in the case of the first phase 1 trial of TGN1412 (anti-CD28 mAb)^66–68^. Recent evidence showed that high-dose IVIG treatment protects from experimental autoimmune encephalomyelitis [EAE], a widely used mouse model for MS, if administered during disease induction, probably by binding to mycobacterial antigens in Freund’s complete adjuvant which is required to facilitate active induction^39^. In contrast, IVIG even exacerbated EAE symptoms if applied 7 days after disease induction^39^. Responses to IVIG in a mouse model of ITP was dependent on strain differences^19^. As pointed out by Mestas and Hughes, it is imperative to understand the potential limitations of extrapolating data from mice to humans, especially where complex multicomponent processes differ^59, 64^. Inherent challenges working with IVIG are imposed by its pleiotropic pharmacodynamics actions^17, 19^ and its xenogeneicity in mice that is associated with gain- or loss-of-function effects^7^. A better understanding of the limitations of current models for IVIG will lead to more caution in interpreting preclinical data obtained in mice^17, 39, 60–63^, and to an improved study design of *in vivo* studies, e.g. with use of humanized mice that are increasingly being used in specific areas of immunology^69, 70^. However, while humanized mice display an interesting alternative to classical mouse models in many immunological aspects, the granulocyte fraction in such models is underrepresented and only accounts for about 3% of the leucocytes^71^, thereby impeding the investigation of neutrophil-driven effects. Accordingly, in regards of granulocyte biology, there is a need of refined humanized mouse models with adequate granulocyte fractions. Significant differences between mouse and human immunology have evolved as a tribute to evolutionary divergence of the species between 65 and 75 million years ago, life in different ecological niches, and differences in size and lifespan^59^. While well-designed animal studies may have value in the identification of potential mechanisms, it will remain key to verify such effects in a human system^7^, and to note species-related differences, when they occur^59^. The findings of our study indicate the need to establish experimental systems that take into account the divergent species-related effects of IVIG on neutrophils.

## Materials and Methods

### Material

The polyclonal IgG preparations used in this study were Privigen, Hizentra, Sandoglobulin (all CSL Behring, Bern, Switzerland) and Immunovenin-Intact (BulBio-NCIPB, Sofia, Bulgaria). Vehicle (250 mM proline) control for Privigen was used to exclude potential IgG-independent effects (Supplementary Fig. 8). Gamma-Venin, a clinical preparation consisting out of F(ab’)_2_ fragments from pooled human plasma IgG, was from Ceneton Pharma, Wien, Austria. Polyclonal pooled mouse serum IgG was from MP Biomedicals (Santa Ana, CA, US). Anti-human IgG monoclonal antibody (mAb) (clone HP-6043-Biot), recognizing all IgG subtypes, and streptavidin–Alexa 633 as well as goat-anti mouse IgG (Alexa Fluor 633) were purchased from Invitrogen, Life Technologies.

To obtain Fc fragments from human IgG, the Streptococcus pyogenes endopeptidase Ide-S (also called Mac-1) was used^72^. Therefore, the cDNA of Ide-S was cloned into the pET28a (GE Healthcare, Little Chalfont, US) expression vector. The expression of the resulting HIS-tagged protein was induced in BL21 *E. coli* (NEB) using 0.1 mM IPTG for 3 hours at 37 °C and the protein was purified using HISTrap HP columns (GE healthcare Little Chalfont, US) according to the manufacturers recommendations using Äkta prime plus (GE healthcare Little Chalfont, US). To obtain Fc fragments of IgG, 300 mg IVIG (Privigen) was incubated with 2 mg Ide-S for at least 8 hours. Digested IVIG was applied to a HiLoad 26/60 Superdex 75 prep grade column (GE Healthcare Little Chalfont, US) and the fractions containing Fc were collected, concentrated (using Amicon Ultra 15 ml centrifugal filter units; Millipore), sterile filtered and stored at 4 °C until use^11^. For quality control, a coomassie stained SDS-PAGE under reducing conditions (5 µg/lane loaded) is shown in Supplementary Fig. 7A. In addition, a size-exclusion chromatography was performed to verify the integrity of the Fc fragments after IdeS-digestion (Supplementary Fig. 7B).

### Cell isolation and cell culturing

Human blood was taken from healthy individuals following informed and written consent in accordance with the Declaration of Helsinki. All experimental protocols were approved by the local institutional and/or licensing committees in Bern and Zürich (Kantonale Ethikkomission; Cantonal Animal Care Committee), Switzerland, by the University Health Network Animal Research Committee in Toronto, Canada, by the Animal Care Committee at the Institute of Microbiology, Sofia according to the ARRIVE (Animal Research: Reporting of *In Vivo* Experiments), National and European Guidelines and in accordance with the National Institutes of Health guidelines for the care and use of experimental animals. All methods were carried out in accordance with relevant guidelines and regulations.

Human neutrophils were isolated from peripheral blood of healthy donors by density gradient centrifugation, as previously described^73^. Briefly, granulocytes and erythrocytes were separated from peripheral blood mononuclear cells (PBMCs) by density gradient using Pancoll (Pan-Biotech, Aidenbach, Germany). The granulocytes and erythrocytes were treated with erythrocyte lysis solution (155 mM NH_4_Cl, 10 mM KHCO_3_, 0.1 mM EDTA, pH 7.3), resulting in cell populations containing at least 95% neutrophils.

C57BL/6 and BALB/c wild-type mice were maintained under pathogen-free conditions. Mouse bone marrow derived neutrophils were isolated from WT mice using magnetic bead based sorting for GR-1^high^ expressing neutrophils as previously described^40^. Analogous, mouse neutrophils were isolated from mouse peripheral blood. Cells were cultured at 2 × 10^6^/mL in the presence or absence of cytokines, Abs, or both, for the indicated times using complete culture medium for human (RPMI 1640 containing 10% FCS and 200 IU/mL penicillin/100 μg/mL streptomycin; Life Technologies, Basel, Switzerland) or mouse (RPMI 1640/GlutaMax containing 10% FCS and 200 IU/mL penicillin/100 μg/mL streptomycin, 50 μM 2-mercaptoetanol; Life Technologies, Basel, Switzerland) neutrophils. Unless otherwise indicated, cells were stimulated with 20 mg/mL polyclonal IgG preparations or the equimolar amount of Fc and F(ab’)_2_ fragments. Cytokine stimulation with human GM-CSF (25 ng/mL; Novartis Pharma GmbH, Nürnberg, Germany) or mouse GM-CSF (1ng/ml, PeproTech, London, UK) occurred 20 minutes before the addition of polyclonal IgG. Further details are provided below and in Supplementary Material.

### Determination of cell death and apoptosis

Cell death was assessed by uptake of 1 μM ethidium bromide (Invitrogen, Lucerne, Switzerland) and subsequent flow cytometric analysis (FACSVerse; Becton Dickinson Biosciences) as previously described^73^. Redistribution of phosphatidylserine (PS) in presence of propidium iodide (PI; Sigma-Aldrich, Buchs, Switzerland) and DNA fragmentation were assessed by flow cytometry as previously described^73^. Recombinant His6-tagged annexin V-FITC, Fas-Ligand (FasL, at 100 ng/ml) and FasL cross-linker (anti-FLAG at 2 μg/ml) were from Alexis (Enzo) and Sigma, respectively. Anti-Fas/CH11 (MBL international corporation distributed by Lab Fore AG, Nunningen, Switzerland) was used at 20 μg/ml. Anti-Siglec-9 (Becton Dickinson Bioscience, Allschwil, Switzerland) was used at 35 μg/ml in combination with 20 μg/ml unlabeled F(ab’)_2_ fragments of secondary GaM Ab (Jackson ImmunoResearch Laboratories, Baltimore, US). Specific cell death was calculated as previously described^73^:

$${\rm{Specific}}\,{\rm{death}}=({\rm{cell}}\,{\rm{death}}[ \% ]-{\rm{death}}\,{\rm{control}}[ \% ])/(100-{\rm{death}}\,{\rm{control}}[ \% ])\times 100.$$

For light microscopic (Zeiss AX10) analysis, 100 cells were evaluated in respect to their morphology as previously described^29^.

### Mitochondrial Potential

Mitochondrial potential was assessed using TMRE-Mitochondrial membrane potential assay kit ab113852 (Abcam, Cambridge, UK) according to the manufactures protocol. Briefly, freshly isolated human neutrophils were incubated for 5 h with or without polyclonal IgG. Carbonyl cyanide 4-(trifluoromethoxy)phenylhydrazone (FCCP) was added (50 μM) for 10 min at 37 °C as a positive control. To assess mitochondrial membrane potential, the cells were stained for 30 min at 37 °C with 100 nM TMRE and assessed by flow cytometry (FACSVerse; Becton Dickinson Biosciences).

### Glycan array analysis

The glycan microarrays from the CFG (Version 5.1) were prepared from amine functionalized glycan structures covalently coupled in microarrays to N-hydroxysuccinimide–derivatized microscope slides as previously described^74^. In accordance to previous publication^5, 55^, Privigen as well as polyclonal pooled mouse serum IgG were screened at 180 μg/ml. In previous studies, vehicle-dependent effects have been excluded at the same concentration^5, 55^. Data are expressed the mean of relative fluorescence units (RFU).

### Complete Freund’s adjuvant (CFA) *in vivo* priming

In order to prime neutrophils *in vivo*, six-week-old female C57BL/6 mice (Janvier Labs) were injected with complete Freund’s adjuvant (CFA; Difco Laboratories). On the day of injection, CFA was diluted in a 1:1 ratio with PBS by vigorously mixing the emulsion for 15 min via transfer in between two syringes connected to each other by a Luer-Lock connector. Mice were anesthetized by isoflurane inhalation and subsequently injected s.c with 100 µl of the emulsion on both sides of the lateral abdomen using a 24 G × 1″ needle. After 24 h of placing the CFA depots, mice were euthanized by CO2 inhalation. For analysis of peripheral neutrophils, whole blood was immediately collected into EDTA-microtainer tubes (BD Bioscience) via cardiac puncture with a 29 G × 0.5″ micro-fine needle (BD Bioscience). Spleens were carefully removed and collected in 15 ml tubes containing cold R10. Spleens were homogenized by passing them through a 70um cell strainer. Subsequently, erythrocytes were lysed using 150 mM NH_4_Cl, 12 mM NaHCO_3_, 0.1 mM EDTA (pH = 8.0) in _dd_H_2_O. Subsequent neutrophil isolation was performed as previously described^40^. Analogous, neutrophils were isolated from peripheral blood. *In vivo* priming was verified by flow cytometric assessment of CD11b staining. Freshly isolated neutrophils from spleen were therefore stained 30 min at 4 °C with FITC-conjugated anti-CD11b (BioLegend). Mean fluorescence ratio (MFI-ratio) was calculated by dividing fluorescence intensity of anti-CD11b stained cells by the fluorescence intensity of a FITC-conjugated isotype control (BioLegends).

### Statistical analysis

Statistical analysis was performed as indicated in the figure legends using GraphPad Prism (GraphPad Software Inc., version 6.0c). If mean levels are presented, SEM and number (n) of independent experiments are indicated. The *p* values < 0.05 were considered as statistically significant.

## Electronic supplementary material

Supplementary information
