# Supplementary material for: IVIG regulates the survival of human but not mouse neutrophils
Source: Sci Rep. 2017 May 2;7:1296. doi: 10.1038/s41598-017-01404-0 (PMC5430961; doi:10.1038/s41598-017-01404-0)
Supplement: Supplementary file 1 — Supplementary information [file 41598_2017_1404_MOESM1_ESM.doc]

**SUPPLEMENTARY INFORMATION**

**IVIG regulates the survival of human but not mouse neutrophils**

Christoph Schneider,^1^ Simone Wicki,^1^ Stefanie Graeter,^1^ Tankica M. Timcheva,^1^ Christian W. Keller,^2^ Isaak Quast,^2,3^ Danila Leontyev,^4^ Iglika K. Djoumerska-Alexieva,^5^ Fabian Käsermann,^6^ Stephan M. Jakob,^7^ Petya A. Dimitrova,^5^ Donald R. Branch,^4^ Richard D. Cummings,^8^ Jan D. Lünemann,^2^ Thomas Kaufmann,^1^ Hans-Uwe Simon,^1^ Stephan von Gunten^1^

^1^Institute of Pharmacology, University of Bern, Bern, Switzerland
^2^Institute of Experimental Immunology, Laboratory of Neuroinflammation, University of Zurich, Zurich, Switzerland

^3^Department of Immunology and Pathology, Central Clinical School, Monash University, Melbourne, Australia

^4^Department of Medicine, University of Toronto and Centre for Innovation, Canadian Blood Services, Toronto, Ontario, Canada

^5^Department of Immunology, Stefan Angelov Institute of Microbiology, Bulgarian Academy of Sciences, Sofia, Bulgaria

^6^CSL Behring, Research, Bern, Switzerland

^7^Department of Intensive Care Medicine, University Hospital Bern (Inselspital), University of Bern, Bern, Switzerland

^8^Department of Surgery, Beth Israel Deaconess Medical Center, Harvard Medical School, Boston, MA, USA

MATERIALS AND METHODS

**IVIG effects on PMN of BALB/c mice *in vivo***

BALB/c 6-8 week old female mice were purchased from Jackson Lab and held under normal housing conditions. IVIg (Privigen 10%, CSL Behring, Berne, Switzerland) or HSA (Alburex 25, CSL Behring, Berne, Switzerland) were used at a dose of 2-4g/kg, injected peritoneally, 24h prior to blood sampling. 50ul of blood from each mouse was collected from the saphenous vein into EDTA coated microtube (Sarstedt, 16.444.100) for analysis by flow cytometry.

Flow cytometry: Absolute number of neutrophils was obtained by adding an internal counting standard (Trucount tubes (BD340334; BD Biosciences, Franklin Lakes, NJ, USA) or reference beads FITC (BD340486). Whole blood samples were stained with Ly-6G PE antibody (BD561104) to identify the neutrophil population, combined with 7AAD (BD559925) staining to exclude dead cells. Alternatively, we used Annexin V-FITC (BD550474) with 7-AAD staining to estimate early and late apoptosis in blood samples. After RBC lysing and washing, the cells were processed as per the manufacturer’s directions (BD Biosciences) and Annexin-V binding determined by flow cytometry using a FACSCalibur (BD Biosciences), and BD CellQuest™software (BD Biosciences). Gating was done on forward scatter versus side scatter of neutrophils identified using anti-Ly-6GPE antibody.

**Sepsis model**

BALB/c mice were purchased from the Charles River Laboratories (Wilmington, MA, USA) and then bred in the Experimental Animals Facility at the Institute of Microbiology, Bulgarian Academy of Sciences. Mice (female, 7 week-old, 15-17 g weight) were kept under standard conditions of a 12-12 hour light-dark cycle and fed with a laboratory diet (29% protein, 13% fat, 56% carbohydrates) and water ad libitum.

BALB/c mice (n=3/group) were injected i.p. with 200 μl LPS (80 μg per mouse; O11:B5, Sigma-Aldrich) or endotoxin-free PBS. After 1 hour, the mice received i.v injection of 100 μl of IVIg (100 μg per mouse) or PBS. Blood was collected by retro-orbital bleeding technique at 12 hours and by cardiac puncture at 24 hours of LPS injection. Blood was used to measure GM-CSF in serum or to detect neutrophils by flow cytometry.

Flow cytometry: Blood was collected in EDTA tubes (1.8 mg EDTA per ml blood) and immediately used for flow cytometry analysis. Blood samples (50 μl) taken at 12 hours and blood samples (100 μl) taken at 24 hours were added to BD Trucount™ Tubes (Lot 16096; Bead count 47000) and incubated with PE/Cy7 labelled anti-Ly6G Ab (clone 1A8, Biolegend; 0.25 μg/100 μl blood) for 15 minutes, dark, RT. Then 450 μl of BD FACS™ lyse/fix buffer was added for 15 minutes, dark, RT and Ly6G+ cells were enumerated by flow cytometry (BSR II Flow cytometer, BD Diva™ Software). Apoptosis of blood and BM neutrophils were detected by Annexin/PI staining.

ELISA: Serum was prepared from blood samples. The amount of GM-CSF was measured by ELISA using commercial kit with detection limit of 16 pg/ml (Biolegend). The amount of the growth factor was calculated from a standard curve of recombinant mouse GM-CSF. In sepsis, levels of GM-CSF were increased as expected (Suppl. Figure 3). The samples were run in triplicate and data were presented as mean±SEM.

**Pigs**

4 pigs (weight 38-42 kg), sedated with propofol and fentanyl and mechanically ventilated, were instrumented for a sepsis trial as previously described^1^. After a period of eight hours with unresuscitated fecal peritonitis, 10 ml of blood were withdrawn from a carotid arterial catheter. Afterwards, the animals were used for another, unrelated study.

1. Corrêa TD *et al*. Effect of treatment delay on disease severity and need for resuscitation in porcine fecal peritonitis. *Crit. Care. Med*. **40**, 2841-2849 (2012).

**
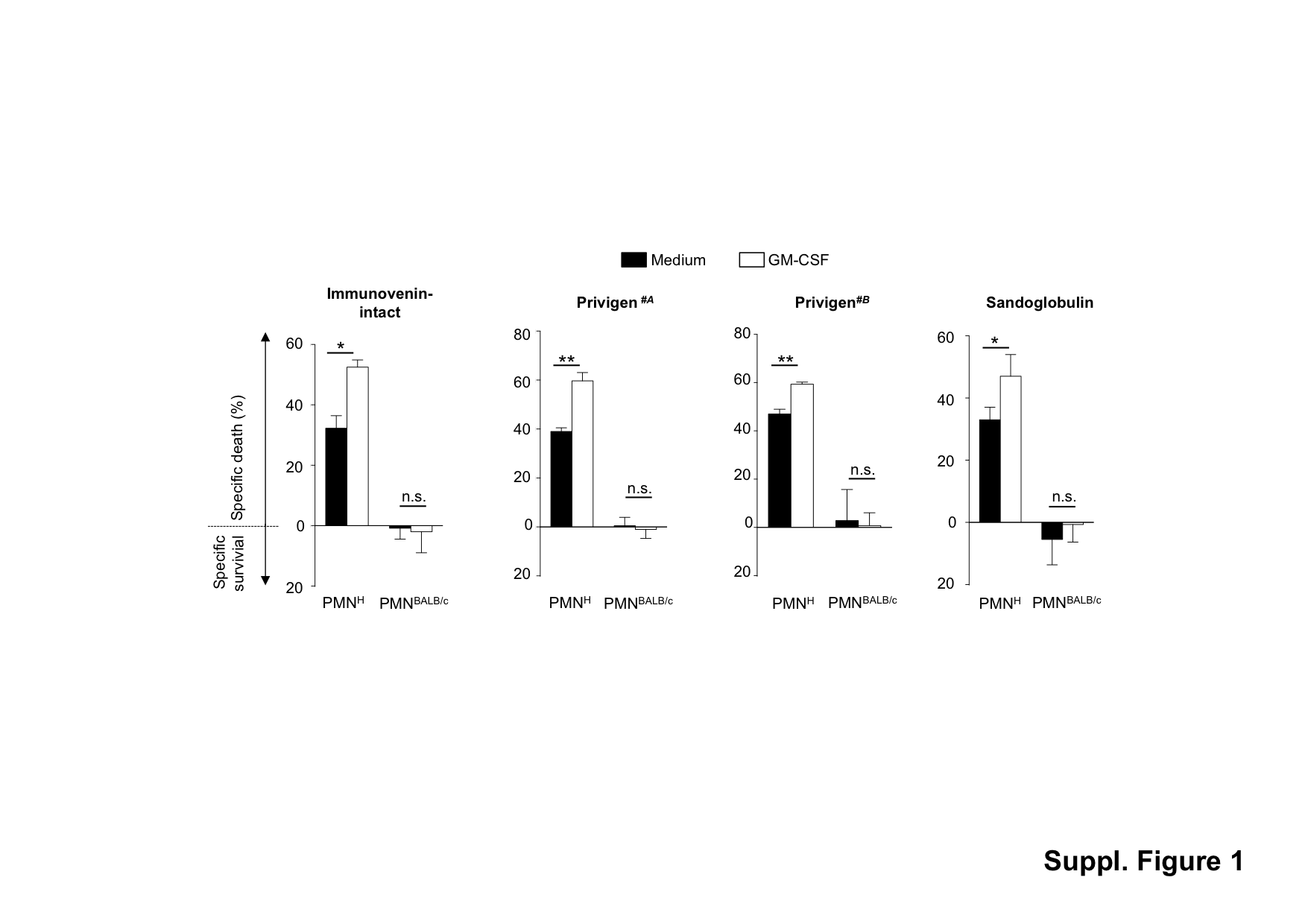
**

**Suppl. Figure 1. IVIG induces cell death in human but not in BALB/c mouse neutrophils**. Death of human (PMN^H^) or BALB/c mouse (PMN^BALB/c^) neutrophils in presence or absence of priming with species matched GM-CSF as assessed by flow cytometric ethidium bromide exclusion assay. Data are representative for 3 independent experiments. *p < 0.05, **p < 0.01, Student *t* test.

**Suppl. Figure 2. Complete Freund’s adjuvant (CFA) activates mouse neutrophils *in vivo*.** Flow cytometric assessment of CD11b expression on mouse neutrophils (BL/6) isolated from spleen with or without 24h *in vivo* immunization with complete Freund’s adjuvant. Indicated are mean fluorescence intensity ratios. Data are representative for 3 independent experiments. *p < 0.05, Student *t* test.


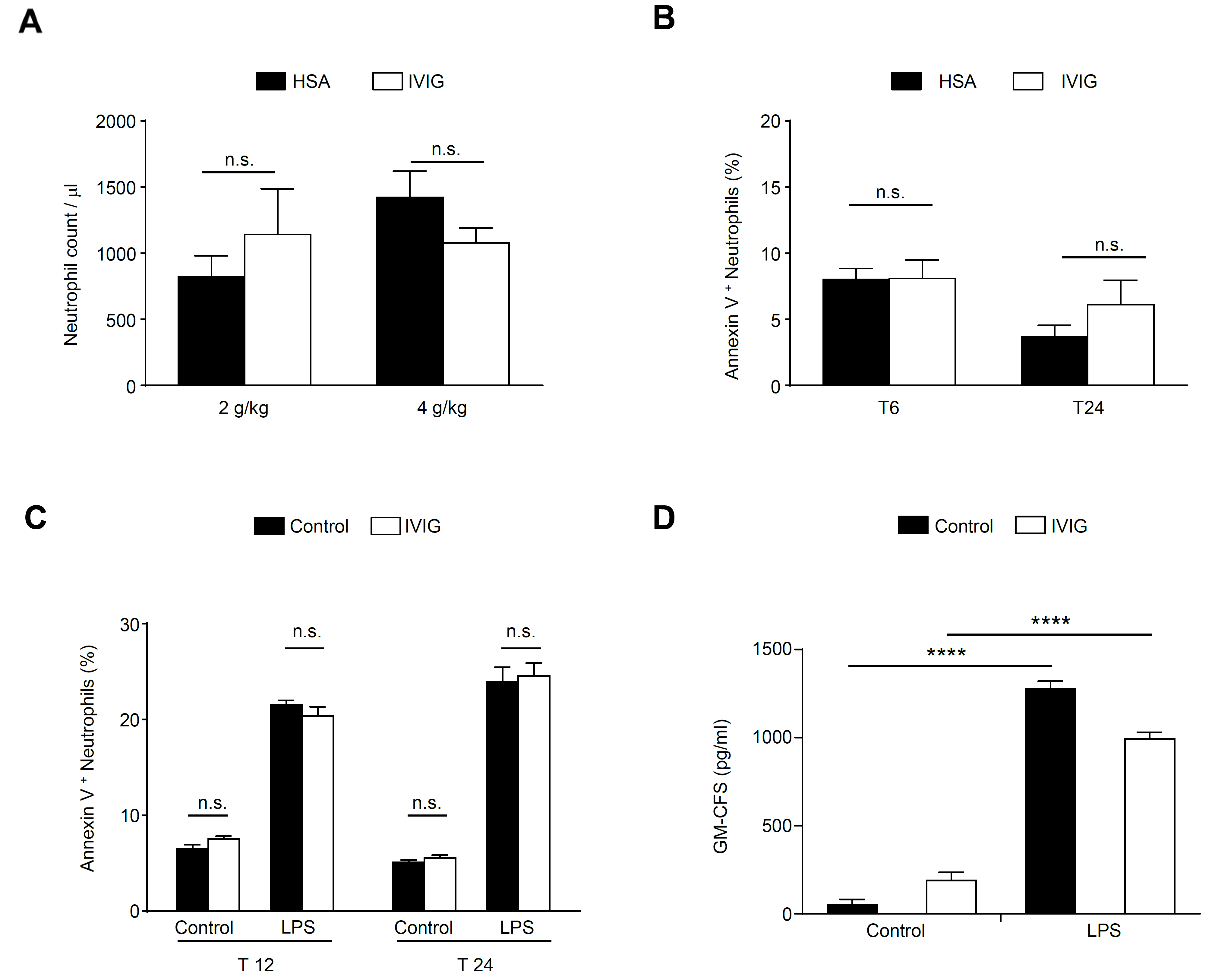


**Suppl. Figure 3. No difference of peripheral blood neutrophil counts and Annexin V binding in healthy or septic mice** **following IVIG administration *in vivo*.** (A) Peripheral blood neutrophil counts following intraperitoneal injection of IVIG or control human serum albumin (HSA) at 4 g/kg in BALB/c mice. (B) Annexin staing of blood neutrophils collected at different time points (6 [T6] and 24 [T24] hours) from untreated or IVIG-treated healthy, wildtype BALB/c mice (4 g/kg). (C, D) Annexin staining of blood neutrophils at different time points (C), and GM-CSF amounts in serum at 12 hours (D), collected from PBS- (control) or LPS-injected BALB/c mice treated with IVIG (4 g/kg). Bars show mean ± SEM. Data are representative of groups of 3 (C), or 5 (A,B) animals. ****p<0.0001, Student t test.

**Suppl. Figure 4. Similar peripheral blood neutrophil Annexin V binding in *ex vivo* cultures in a porcine model of fecal peritonitis.** (D) Flow cytometric ethidium bromide exclusion assay. Viability of pig peripheral blood neutrophils from pigs before and 1 day after induction of sepsis. Results of 24-h cultures in presence of absence of IVIG at 20 mg/ml are shown. Bars show mean ± SEM. Data are representative of groups of 4 animals. *p < 0.05, Mann-Whitney U test (D).


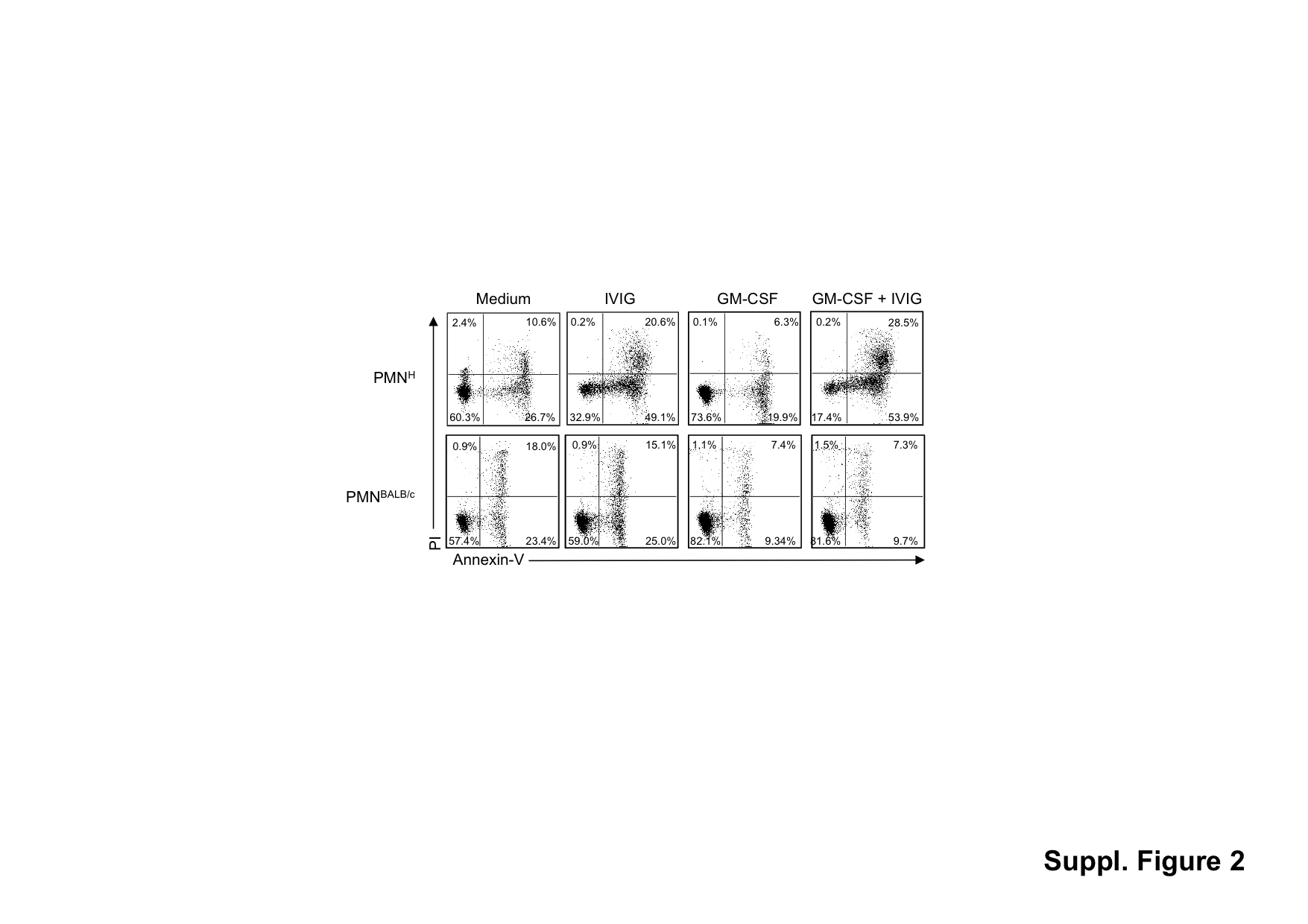


**Suppl. Figure 5. IVIG promotes death of human but not BALB/c mouse neutrophils.** Cell death of GM-CSF primed or unprimed human (PMN^H^) or BALB/c mouse (PMN^BALB/c^) neutrophils following treatment with IVIG as assessed by Annexin V-FITC/PI staining assay. Data are representative for 3 independent experiments.

**Suppl. Figure 6. Confirmation of selective survival regulatory capacity of IVIG in human but not mouse neutrophils by different death assays.** Statistical summaries for Annexin-V/PI staining (A) and DNA fragmentation (B) of human (PMN^H^) or mouse (PMN^BL/6^) neutrophils. Data are representative for 3 independent experiments. *p < 0.05, **p < 0.01, ***p < 0.001, Student *t* test.


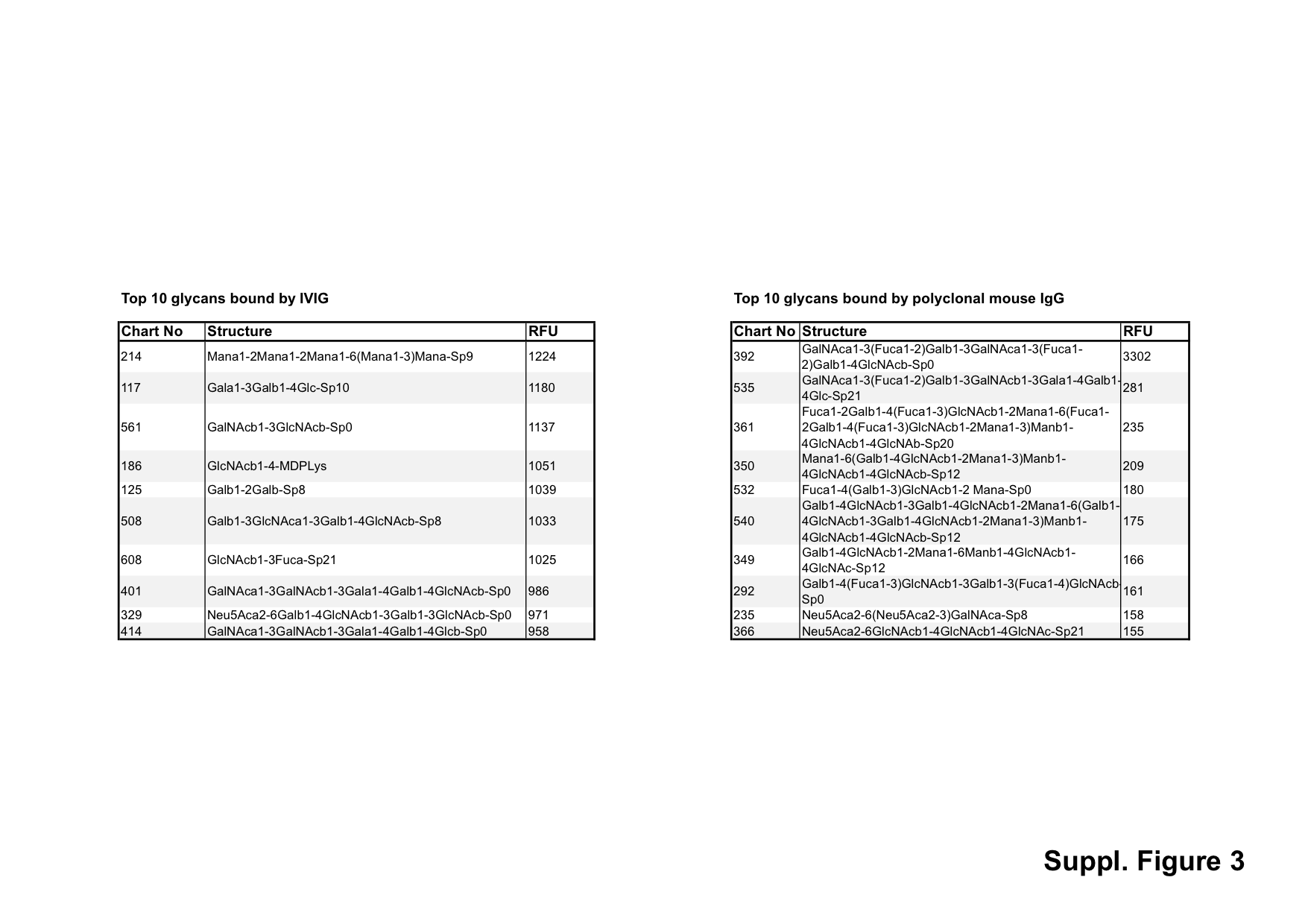


**Suppl. Figure 7.** Carbohydrate-binding profiles of IVIG or polyclonal mouse IgG as assessed by CFG glycan array technology. Results for the top 10 glycans bound ranked according to relative fluorescence units (RFU).

**Suppl. Figure 8. Privigen-induced cell death is independent of its vehicle.** Cell death of GM-CSF-primed or unprimed neutrophils following 24h incubation with Privigen or vehicle as assessed by flow cytometric ethidium bromide exclusion assay. Data are representative for 3 independent experiments. **p < 0.01, ***p < 0.001, Student *t* test.

**
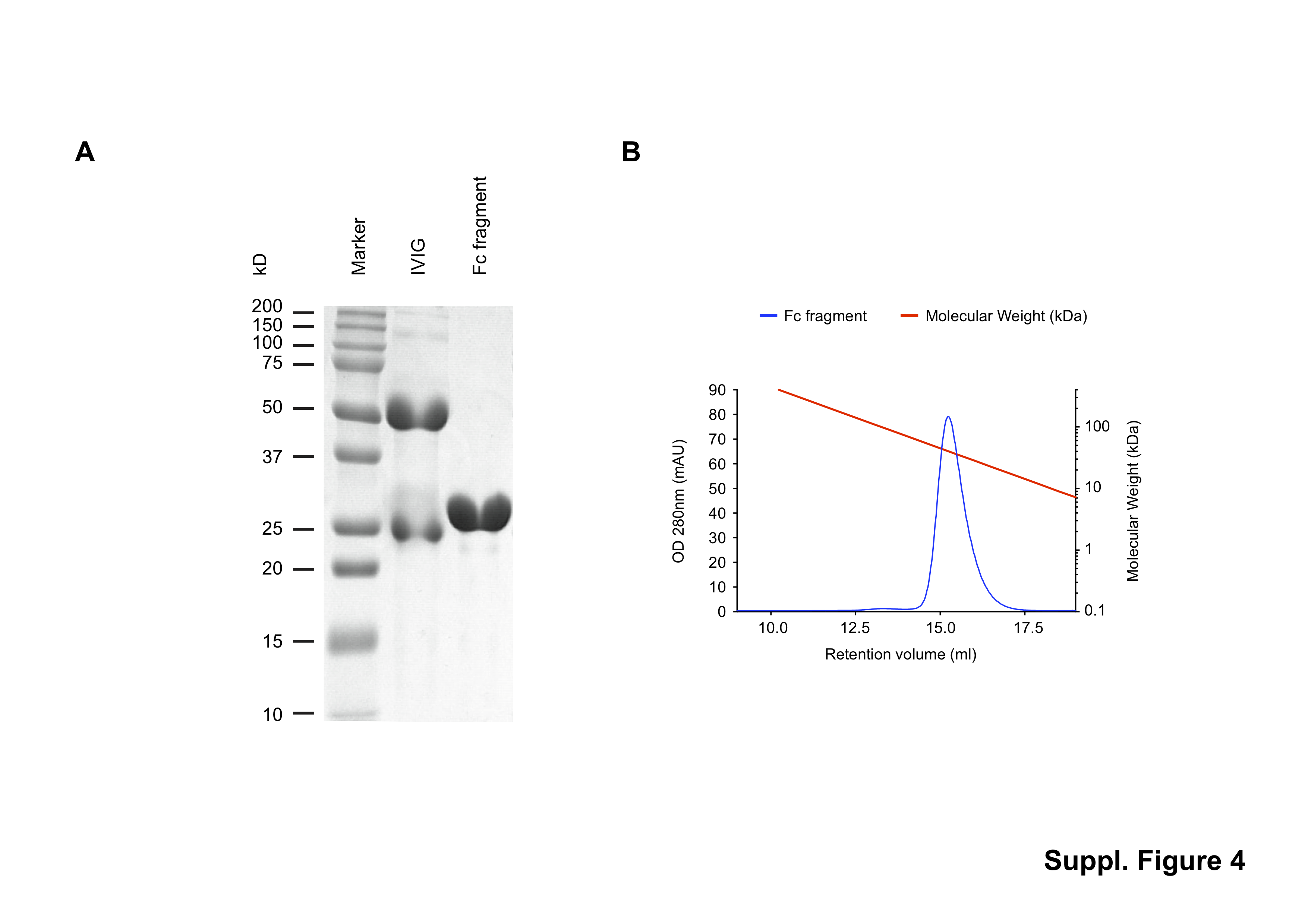
**

**Suppl. Figure 9.** (A) Coomassie stained SDS-Page of IVIG and Fc fragments performed under reducing conditions. 5 μg/lane was loaded. (B) Size-exclusion chromatography of Fc fragments after IdeS-digestion and purification.
